# Research on the Security of IPv6 Communication Based on Petri Net under IoT

**DOI:** 10.3390/s23115192

**Published:** 2023-05-30

**Authors:** Yu Han, Liumei Zhang, Yichuan Wang, Xi Deng, Zhendong Gu, Xiaohui Zhang

**Affiliations:** 1 School of Computer Science, Xi’an Shiyou University, Xi’an 710065, China; 20212060708@stumail.xsyu.edu.cn (Y.H.); 20222060785@stumail.xsyu.edu.cn (X.D.); 2 School of Computer Science and Engineering, Xi’an University of Technology, Xi’an 710048, China; chuan@xaut.edu.cn (Y.W.); xhzhang@xaut.edu.cn (X.Z.); 3 Shaanxi Key Laboratory for Network Computing and Security Technology, Xi’an 710048, China; 4 Hanjiang-to-Weihe River Valley Water Diversion Project Construction Co., Ltd., Xi’an 710024, China; guzhendong@hwrvwd.cn

**Keywords:** IoT, IPv6, NDP, SDN, Petri Net

## Abstract

The distribution of wireless network systems challenges the communication security of Internet of Things (IoT), and the IPv6 protocol is gradually becoming the main communication protocol under the IoT. The Neighbor Discovery Protocol (NDP), as the base protocol of IPv6, includes address resolution, DAD, route redirection and other functions. The NDP protocol faces many attacks, such as DDoS attacks, MITM attacks, etc. In this paper, we focus on the communication-addressing problem between nodes in the Internet of Things (IoT). We propose a Petri-Net-based NS flooding attack model for the flooding attack problem of address resolution protocols under the NDP protocol. Through a fine-grained analysis of the Petri Net model and attacking techniques, we propose another Petri-Net-based defense model under the SDN architecture, achieving security for communications. We further simulate the normal communication between nodes in the EVE-NG simulation environment. We implement a DDoS attack on the communication protocol by an attacker who obtains the attack data through the THC-IPv6 tool. In this paper, the SVM algorithm, random forest algorithm (RF) and Bayesian algorithm (NBC) are used to process the attack data. The NBC algorithm is proven to exhibit high accuracy in classifying and identifying data through experiments. Further, the abnormal data are discarded through the abnormal data processing rules issued by the controller in the SDN architecture, to ensure the security of communications between nodes.

## 1. Introduction

The Internet of Things (IoT) [1] technology was proposed by the International Telecommunication Union (ITU) and is the core concept of the information industry revolution and the industrial revolution. The continuous development and innovation of IoT-related technologies have changed the production modes of traditional industries, giving rise to a large number of smart devices and service models, which include IPv6 [2] communication, cloud computing and sensing devices. The IoT system is built with numerous nodes and with management mechanisms including addressing and routing to support the communication. The increasing number of nodes poses the problem wherein the traditional IPv4 can no longer handle the demands of the IoT for network addresses. Compared to IPv4 addresses, IPv6 addresses contain 128 bits. While this solves the address scarcity problem that exists in the IoT, it also introduces new security threats. First of all, as a large number of IoT devices access the Internet in the process of IPv6 communication, it is easy for security risks to arise, such as the remote control of devices and leakage of sensitive data. Second, security issues in stateless address allocation during IPv6 communication, insecure authentication during address resolution and DDoS attacks faced by global anycast technology are encountered. All of these can affect the security performance of the IoT. In addition to allowing more devices and users on the Internet, the IoT also provides many additional features, such as improved efficiency in routing traffic and increased flexibility in address assignment  [3]. When two hosts start communication, it is necessary to know the MAC address of the communicating host. In IPv4 communication, the Address Resolution Protocol (ARP) [4] is required to look up the MAC address for a given IP address. Meanwhile, in IPv6 communication, the NDP [5] protocol is responsible for the lookup of the MAC address, which lacks authentication. Therefore, any NS or NA messages on the same link can be manipulated, which may lead to the launch of a DDoS attack [6]. In a DDoS attack, an intruder controls a computer to intercept the communications between hosts through various technical means. They may obtain the source IP addresses of the communicating hosts, and then attack the victim hosts with numerous NS messages or NA messages [7]. Then, they can further overflow the neighbor cache table, affecting the normal communication of the network. In response to this situation, there are many passive solutions proposed to detect, mitigate and prevent attacks, including the use of static IP-MAC address assignment [8] and the use of hardware [9] and software to monitor the changes of IP-MAC pairs [10]. The main drawback of these passive solutions is the lack of dynamism, scalability and false intelligence. Some active solutions provide detailed techniques and solutions, such as the IDS actively sending probe packets to hosts in the link to eliminate flaws. There are also academics who suggest using Secure NDP (SEND) [11] and Trust NDP [12], but these techniques either require a rational tradeoff between time and bandwidth consumption or are subject to DDoS attacks because of their design. In this study, the SDN framework is built to ensure normal traffic communication effectively by issuing rules through the controller so that abnormal data traffic can be discarded.

Petri Net [13] is a modeling and analysis tool with rigorous mathematical definitions and powerful graphical representations to describe the static structure and dynamic behavior of the simulation process. Cyber attacks and defence within Petri Net have been widely studied, but there is still little research utilizing fine-grained analysis and modeling for a single attack.

The software-defined network [14] (SDN) is a novel network architecture designed to achieve higher flexibility and manageability in the network, as it decouples the control plane and data plane. The whole architecture shows the characteristics of NC separation. There are some available solutions for DDoS attack detection under the SDN architecture, but these are mainly for DDoS attack detection and prevention under the IPv4 protocol.

In this study, we aim to detect and prevent DDoS attacks under IPv6 communication by modeling Petri Net on the SDN architecture. The main contributions are listed as follows.

(1) A Petri Net model of an NS flooding attack under IPv6 communication is proposed. Compared with other studies, the NS flooding attack towards IPv6 address resolution is described in detail from the underlying principle of the attack. We judge the state of communication by using the states of nodes. This paper models the NS-DDoS attack on the address resolution process under IPv6 communication by using the Petri Net model, where Place represents the attack state, Transition represents the attack behavior and Arc represents the change between states. The NS-DDoS attack behavior is modeled using the Tina tool by setting up the initial token and analyzing the model by observing the movement of the token in the model.
(2) When using the SDN NC hierarchical structure, NS flooding attacks often target the neighbor cache table of nodes. This is because NDP is insufficient for message verification. Meanwhile, in the SDN architecture, the flow table is used to collect data, and the controller sends down rules for processing, which is safer and swifter to process against abnormal attack packets.
(3) Based on the anomaly detection of Naïve Bayes Classification (NBC) [15], we introduce NBC to the SDN architecture. Through the analysis of the IPv6 source address growth rate, flow table growth rate, MAC address growth rate and other characteristics, the attack data are effectively detected and the controller issues packet loss rules, thus effectively ensuring the secure communication of IPv6. In this paper, through the training of three classification algorithms and the comparison of several indicators, the results prove that the anomaly detection and defense based on the NBC on the SDN architecture can effectively guarantee normal communication under the IPv6 protocol.

This paper proposes an attack model using Petri Net for NS flooding attacks under IPv6 communication and proposes a corresponding defence model under the SDN architecture, and then effectively maintains the network security under IPv6 communication. The rest of the paper is organized as follows. The first part introduces the research background of this paper. The second part introduces the work related to the NS flooding attack under the NDP protocol, Petri Net and the SDN architecture. The third part introduces the NS flooding attack and modeling under IPv6 address resolution. The fourth part introduces DDoS defence and Petri Net modeling based on the random forest algorithm under the SDN architecture. The fifth part introduces the experimental environment. The sixth part presents the experimental analysis. The seventh part concludes and summarizes the research of this paper.

## 2. Related Work and Background

As an important part of the IPv6 protocol, the security of the NDP protocol has received extensive research attention. Anbar M [16] studied the attacks under the NDP protocol, and he divided NDP attacks into two categories: one is MITM [17] attacks, and the other category is DDoS attacks. Zhang [18] et al. analyzed NDP and SEND’s security. They summarized the NDP protection methods and the latest progress in enhancing NDP security, and experimented by introducing the SEND mechanism, which can solve many security problems in NDP. However, the SEND mechanism is not widely used. Arjuman N C. [4] introduced an authentication mechanism to improve the security of NDP. The proposed framework uses the multicast key management protocol as an application layer key management scheme to solve the multicast problem in neighbor communication. The framework introduces Internet Protocol Security (IPsec) [19], Authentication Header (AH) and Media Access Control (MAC) address options in NDP for the authentication of communication packets to prevent attacks on forged ND messages. The improved NDP security policy can effectively defend against NDP security attacks, such as SYN flooding, forged prefix address attacks and ARP spoofing. Ahmed K. Al-Ani [20] proposed a prevention technique, namely Match Prevention, that secures target IP addresses and exchange messages (i.e., NS and NA). A. Q. Moghadam [21] proposed a method that uses entropy to detect the randomness of flowing data. This method can rapidly detect TCP SYN flood attacks. Although this method is effective, when Robinson [22] and Wang et al. used it to detect DDoS attacks, they discovered that it was cumbersome to implement. Various researchers have introduced machine learning as a DDoS detection algorithm. F. Ouakasse [23] et al. used SVM as a DDoS detection algorithm. Although the algorithm performed well on the KDD99 dataset, its performance in the actual environment is unknown. S. Dong [6] used the improved KNN algorithm to detect DDoS attacks, which had high accuracy. However, it cannot be applied to a real environment.

### 2.1. NDP

NDP represents multiple messages and processes used to establish communication between nodes, routers and hosts located in the same IPv6 network. To achieve its functionality, NDP uses the following ICMPv6 [24] messages.

Neighbor Solicitation (NS).This is an alternative to the ARP protocol in IPv4 and uses NS messages to determine the link-layer address of the neighbor, to verify the address during the Duplicate Address Detection (DAD) process or to verify the reachability status of the neighbor.

Neighbor Advertisement (NA). This involves announcing changes to host MAC and IP addresses or responses to NS message requests.

Router Solicitation (RS).The host queries the RS message to locate the router on the local link network and prompts the router to respond immediately, Reply (RA).

Router Advertisement (RA).RA messages are periodically sent by routers or in response to RS requests. Routers use RA messages to notify other nodes of their presence on the network, and to send system parameters such as MTU, network prefix, hop count, etc.

Redirect (RM). RM messages are used to redirect traffic from one router to another.

### 2.2. Petri Net

Petri Net is a general-purpose discrete-event modeling tool used to model, analyze and describe control and information flows in discrete-time or distributed systems with asynchronous and concurrent activities. Petri Net can both describe the structure of a system and model its operational state. As a modeling tool, the Petri Net model provides an intuitive way to understand systems with dynamic behavior. It has a wide range of applications in system modeling and is characterized by strict formal definitions, rich expressiveness and intuitive pictorial descriptions. Petri Net is suitable for describing mesh models of asynchronous concurrent systems, both to describe the structure of the system and to model its operation. Classical Petri Net models are simple process models consisting of two types of nodes, Place and Transition, as well as directed arcs, tokens and other elements [25].

 **Definition 1.** *A triple PN=(P,T,F) is called a Petri Net if it satisfies the following conditions:*

 *(1)*  *P is the finite set of Places and T is the finite set of Transitions.*
 *(2)*  *Among them, P≠ϕ, T≠ϕ and P∩T=ϕ.*
 *(3)*  *F=(P×T)∩(T×P) represents the flow relationship of PN.*

In this paper, through a microscopic analysis of the NS flooding attack in the address resolution process, an attack model and a defense model are established using Petri Net. We address the limitations of the traditional model and describe the attack process and defence mechanism in detail. The security of IPv6 communication is ensured effectively.

### 2.3. SDN Architecture

**Figure 1:**
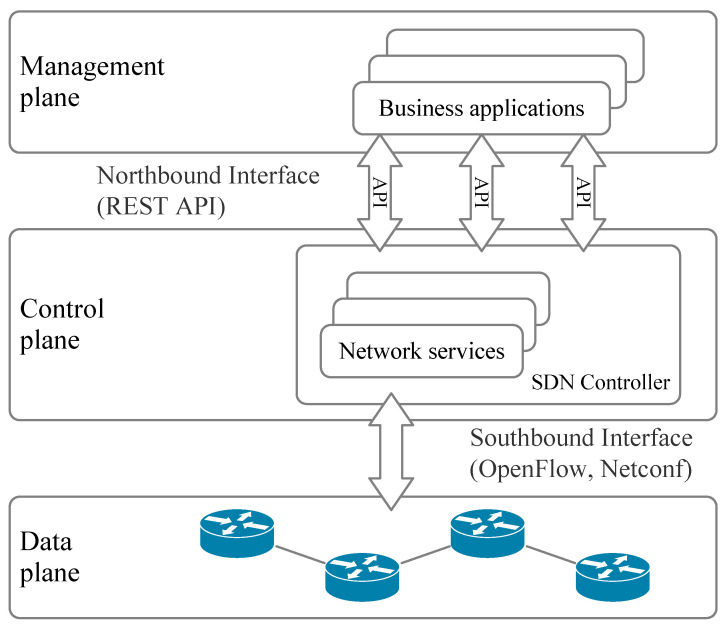
SDN architecture.

The software-defined network (SDN) is an emerging network architecture whose core idea is to decouple the control layer from the data layer to achieve the centralized control of hardware devices. The controller performs the management, control and decision-making processes of the switch, and the switch is only responsible for data forwarding, making the network structure flexible and efficient. The concept of OpenFlow technology was first proposed by Professor Nick McKeown of Stanford University [26]. Using OpenFlow as the southbound interface protocol for SDN enables effective interaction between controllers and switches. OpenFlow is currently the most widely used southbound interface protocol for SDN. The controller controls the forwarding mechanism of the switch through the formulation of flow table rules. Certain blocking of DDoS attacks is performed to ensure normal communication, as shown in Figure 1.

## 3. NS Flooding Attack with Petri Net Model

### 3.1. Principle of Attack

When a source node needs to communicate with a target node on the same link, the source node needs to know the MAC address of the target node, and the nodes on the link use NS messages and NA messages to create a link between the two nodes. Each node has a neighbor cache table. When the nodes need to communicate with each other, they must know the MAC address of the corresponding node. The source node requests the target node’s MAC address by sending an NS message to the multicast address of the requested node. This message type is 135 and includes the source address, destination address and link layer address of the source address itself. After the target node receives the NS message, the target node first extracts the source address and the message. The link layer address in the file option is added to or updated in the local neighbor table to form a mapping relationship. Then, the requested node sends NA packets with the destination address as the unicast address.

**Figure 2:**
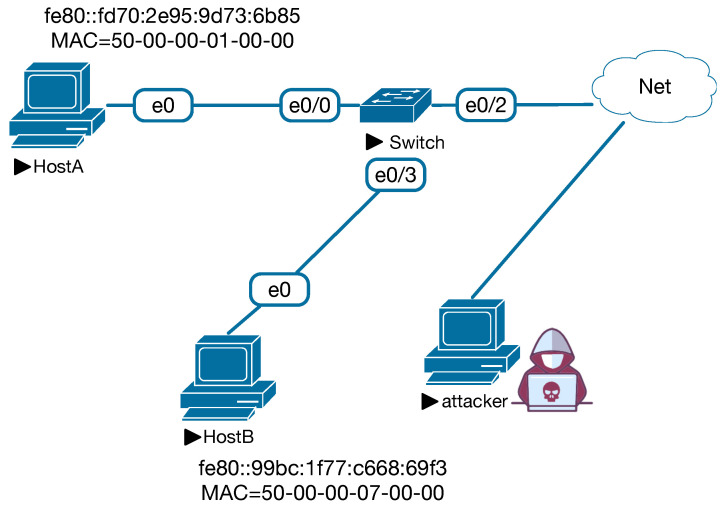
Attack environment setting.

Host A needs to resolve the link layer address of Host B before sending a message to Host B. The nodes on the link use the neighbor request message and the neighbor advertisement message to create an IP-MAC mapping relationship in the neighbor cache table, so Host A sends an NS message where the source address is the IPv6 address of Host A, the destination address is the multicast address of the requested node of Host B and the destination IP to be resolved is the IPv6 address of Host B. It also carries its own MAC address. Moreover, in message sending, a record of the recipient IP is created in its cache, and it sets its status to incomplete. When Host B receives the NS message, it verifies whether the NS message is received by verifying the multicast address and IP address of the requested node. If successful, Host B will extract the source address and the source link layer MAC address in the message option, form a mapping relationship, add or update it in the local neighbor table and then carry its own MAC address and IP address to send the NA message with the destination address as a unicast address to the requester. After Host A node receives the NA message, it updates its own neighbor table according to the IP-MAC mapping relationship based on the MAC address carried in the option. Its status is Reachable. While awaiting NA messages, Host A updates its own neighbor table status to Stale if it times out. Because the NS message is sent out as a multicast, other nodes on the multicast can also receive the relevant information and know the MAC address of the source node, and the destination node enables the request flag S when responding to the NA message to the source node, so when the attacker “sniffs” the NS message, obtains the IPv6 address of the source node and performs a DDoS on the address, it obtains the address of the source node and performs a DDoS attack on this address. This causes the neighbor cache table to overflow, and Host A and Host B cannot communicate normally. This is shown in Figure 2.

### 3.2. Attack Process

When Host A pings Host B, the attacker obtains the IPv6 address of the source node by sniffing the multicast NS messages, and then launches an NS flooding attack on the source node using the THC-IPv6 [27] tool.

The sniffer code is as follows.


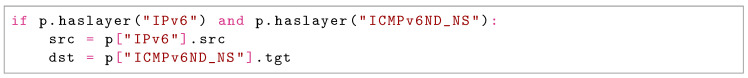


### 3.3. Petri Net Model Based on NS Attacks

The principle of the NS flooding attack in the Petri Net model is as follows. The source node first checks the neighbor cache table. When no match is found, the source node sends an NS packet to the target node. After it receives the NS packet, the source node then returns a unicast NA packet to the source node. The target node updates its neighbor cache table, and then the two nodes communicate. In the NS flooding attack, the attacker uses a sniffing code to obtain to the source node’s IPv6 address. Then, they generate multiple random IP addresses to request NS packets from the source node, causing the neighbor cache table to overflow. Through microscopic analysis of the attack process, a Petri Net attack model diagram reflecting the NS flooding attack is established, which can map the whole process of the NS flooding attack.

**Figure 3:**
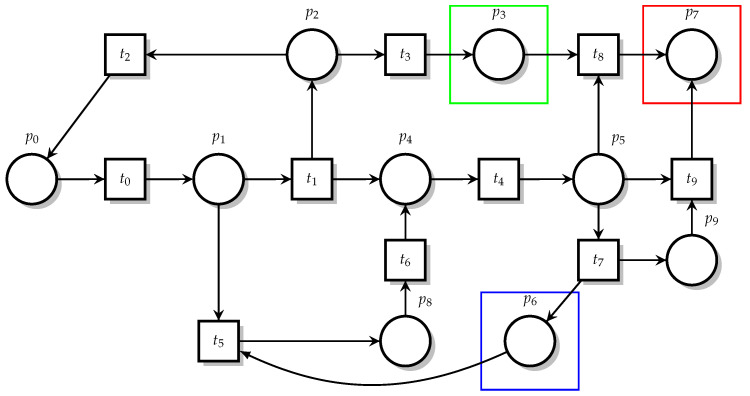
NS flooding Petri Net diagram.

The mapping relationships when building Petri Net models are shown in Table 1. In the formal modeling process, various states are included, such as the normal state, communication state, illegal state, etc. The main operations that exist include sending NS packets and NA packets, sniffing NS messages, attacking machines for flooding attacks and many other operations. In this model, Place mainly represents the various states in the protocol, Translation represents the various operations in communication and Token represents the number of communication requests or the number of attacks, which is formulated according to the actual situation. The Petri Net model is built according to the rules. When the model is run, a mutation occurs only when the input place contains at least one token and the arc weight is 1. When the arc weight is greater than 1, each input place in the variation contains at least as many tokens as the arc weight, the output place is subtracted by 1 and the input place is increased by 1. Only then can the transformation take place. According to the Petri Net modeling rules, the Petri Net model under an NS DDoS attack is established. Place denotes the state of the message and is represented by a circle, while Transition denotes the change in the message state and is represented by a square, and the initial Place token value can be set according to the communication state. This is shown in Figure 3.

The meaning of Place and Transition under NS flooding is shown in Table 2. By analyzing the principle of the NS flooding attack and address resolution protocol, we build a Petri Net model of the NS flooding attack. From the table, we can see that $P_{0}$ belongs to the source node and sends NS messages to the multicast node in sending messages to the $P_{4}$ target node. After checking that there is no matching information in the neighbor cache table, $P_{3}$, as the attacking machine, sniffs the NS messages of the multicast node and then performs an NS flooding attack on the source node. $P_{7}$ belongs to the damaged state, and the result is that IPv6 cannot communicate normally. One reason is that when its own neighbor cache table is full, it is in the $P_{9}$ state and $P_{5}$ is still receiving new messages, and the other is that the $P_{3}$ attack machine can launch an attack on the source node, causing the communication to be blocked.

After describing and analyzing NS-DDoS attacks on Petri Net, it can be seen that in an NS-DDoS attack on a node, the attacker obtains the IP address of the source host by sniffing the NS messages sent by the source node to perform a DDoS attack on the source node. In the Petri Net model, the Place and Transition descriptions are used to depict the attack flow and state, as well as the changes between events, in detail. The researcher can understand and detect the NS-DDoS attack in an effective manner and judge the attack behavior based on the changes in the model.

In IPv6 communication, the attack principle and the attack behavior need to be combined so that the attack can be modeled and analyzed as a Petri Net model. This paper offers only a qualitative analysis of the attacks on Petri Net, and a quantitative analysis is not possible. At the same time, research on the dynamic generation of Petri Net models by attackers is still in progress, and Petri Net models cannot be dynamically generated or built on the system.

## 4. Protection against NS Flooding Attacks Based on SDN Architecture

NBC is a common classification algorithm. It mainly calculates the corresponding posterior probability based on the prior probability and conditional probability of the features. In the process of cyber-security data analysis, the NBC algorithm is often used to process the data. In [28], the authors developed an active machine learning algorithm based on NBC classification. The aim was to discover unknown Android malware through static analysis. High accuracy of detection is demonstrated. The authors of [29] propose a new anomaly intrusion detection system (IDS) by combining NBC detection algorithms. Meanwhile, in [30], the authors used principal component analysis and linear discriminant analysis algorithms to reduce the dimensionality of the data. Then, they combined the method with NBC and proposed the PCA-LDA-NBC algorithm. The authors of [31] used a combination of Snort and NBC to propose an intrusion detection system (IDS) based on a cloud computing infrastructure. In this article, we describe the principles of NBC by learning about the attack traffic of NS-DDoS. We calculate the prior probability of its attack traffic and normal traffic. The conditional probability is calculated for the features in the traffic. Then, it is substituted into a Bayesian formula for calculation, so that the traffic can be effectively detected and classified.

The traffic passing through the switch is classified and the packet is dropped by calculating the prior probability and conditional probability of the NBC, where the code is entered as follows.


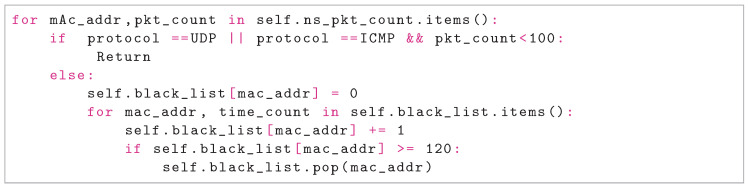


### 4.1. Package Rule Processing

The attacking hosts in this study use the THc-IPv6 tool to attack normal hosts, and we randomly generate the source IP addresses of the NS messages. The Bayesian classifier is used to distinguish the abnormal and normal traffic. Next, the controller issues rules through the flow table for the switch to discard the attacking packets. Meanwhile, in the packet processing rules, the packets are discarded after the abnormal traffic is classified by the Bayesian model. Thus, the controller can issue forwarding rules to the switch through the formulation of flow table rules. Thus, the switch discards the detected attack packets and the source MAC class information of the attack packets is dropped to ensure normal communication under IPv6.

### 4.2. Petri Net Model Based on NBC Detection

**Figure 4:**
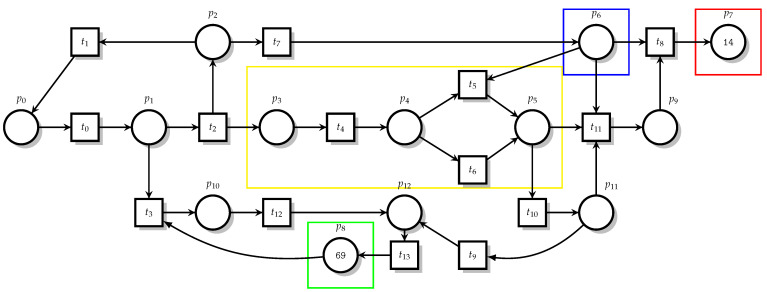
Defending against Petri Net.

In an NS flooding attack, the NDP protocol of the attacking machine is insufficient for message verification and to address the overflow of the neighbor cache table. For this problem, we propose a Bayesian algorithm abnormal traffic detection defense model based on the SDN architecture, shown in the yellow area in Figure 4. In this architecture, the traditional switch directly forwards traffic communication, and the controller assigns rules to the switch and controls the forwarding of traffic. As seen from Figure 4, $P_{3}$ is the initial flow table; every unmatched message will be sent by the flow table $T_{4}$ packet to the controller, and $P_{4}$ controller will send a packet-out message after receiving the message, instructing the SDN controller to request the current traffic statistics by periodically sending OFP flow stat request messages to the switch. Then, the switch reads the relevant statistics from the flow table and replies to the controller, completing the collection of the flow table information. $T_{5}$ is the collection of traffic, including traffic generated by the attacking machine, normal traffic $T_{6}$ with packet-in forwarding and normal communication. By referencing rules from the flow table, attack traffic can reach $P_{5}$, which contains algorithms and packet loss. Thus, the switch has forwarding conditions and restrictions only when the algorithm does not identify abnormal traffic. Next, the source host sends an NS flooding message, which will cause abnormal communication $P_{7}$; otherwise, it passes into the normal state $P_{8}$. When they start to communicate again, the flow table has a match for the message, so it will communicate with the node directly. This is shown in Figure 4. The NS flooding defence against Petri Net’s Place and Transition features is shown in the Table 3.

## 5. Experimental Environment

### 5.1. Experimental Environment

**Figure 5:**
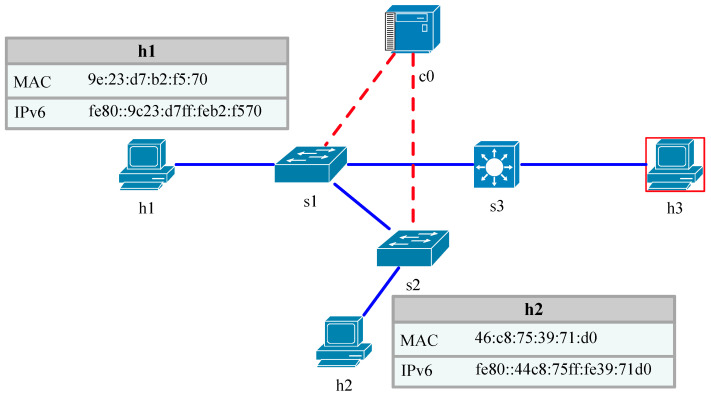
IPv6 communication defense diagram under SDN.

In this study, the NS flooding attack network is built in the EVE-Ng [32] environment, as shown in Figure 2. Host A and Host B are normal hosts, and Kali is connected to the external network as the attacking host. The NS flooding defense environment is built on Ubuntu Linux with the SDN architecture, where the SDN architecture includes the RYU [33] controller, Mininet emulation and an OpenFlow flow table, and the switch uses Open vSwitch. In the defense emulation environment, H1 and H2 are normal hosts, H3 is the attacking host that initiates the sniffing message operation and flooding attack on H1, which sends the Ping command, and the generated flow table entries are extracted from switch S1 through controller C0 and analyzed and re-issued as rules to the switch. This is shown in Figure 5.

### 5.2. Data Source

The data in this article were collected by ourselves. H3 performed an NS flooding attack on H1 using the THC-IPv6 tool, and later captured the information using the Wireshark tool. If the reader would like to access the data presented in this paper, they may visit www.kaggle.com/hanyu9337/ns-flooding-dataset, accessed on 11 October 2022.

The dataset collected by the THC-IPv6 tool contains Smurf attacks, Flood solicitate6 attacks, Flood router attacks, Flood advertise6 attacks and other attacks. The dataset collected by the THC-IPv6 tool contains Smurf attacks, and these features’ fields contain core feature fields for time or different protocol types. Table 4 describes the specific meanings of these feature fields.

The above dataset contains all attack features from the THC-IPv6 tool, including public features and DDoS attack feature sets. This paper focuses on NS-DDoS attacks in IPv6, so the feature sets described in this paper are related to NS-DDoS attacks.

In total, the collection includes 111,650 data items for the NS-DDoS attack. The attributes of the dataset include time, source IP, destination IP, protocol, length, message and label. We determine the abnormal traffic and normal traffic by determining the source IP and destination IP, and by counting the packets per unit time.

### 5.3. Feature Extraction

(1) Source IP Growth Rate Equation (1)
  (1) $$\mathrm{SIGR}=\frac{\mathrm{SourceIPNum}}{\mathrm{Interval}}$$

SourceIPNum is defined as the number of different source IP addresses in a certain time; Interval is the time interval. During NS flooding, the attacking host generates a large number of random and fake IP addresses, which causes the source IP to grow significantly faster.

(2) Port Growth Rate Equation (2)
  (2) $$\mathrm{PGR}=\frac{\mathrm{DifferentPortsNum}}{\mathrm{Interval}}$$

In the normal state, the service port growth rate is in a relatively smooth range, while, when a DDoS attack occurs, the attacker will generate random ports for connection, so the port growth rate will increase sharply compared to the normal state.

(3) ICMP Message Growth Rate Equation (3)
  (3) $$\mathrm{ICMPGR}=\frac{\mathrm{ICMPNSNum}}{\mathrm{Interval}}$$

For IPv6 under address resolution, the system will send ICMP-type NS packets. Under NS flooding, the NS unit time interval is an important feature.

## 6. Analysis of Experimental Results

The processing of the datasets is mainly used to distinguish the traffic as normal traffic and abnormal traffic and to perform binary classification on the original datasets. The most common linear classifiers include NBC and SVM [34] algorithms. NBC is suitable for dealing with text-based classification, where the attack dataset features contain a certain amount of text, which is more effectively processed using NBC. SVM is a machine learning algorithm used to solve the binary classification problem, which has certain advantages in dealing with small sample datasets and has a strong generalization capability. In addition to linear classifiers, for multiple features in the dataset, we consider the non-linear classifier RF for comparison with linear classifiers.

In the first experiment, we use the experimentally collected datasets and analyze them using the accuracy, recall and precision, F1 score, training time and other metrics.

True positive (TP) indicates the number of samples for which the model correctly detects the attack stream sample data as attack stream data. True negative (TN) indicates the number of samples for which the model detects the normal stream sample data as normal stream data. False positive (FP) indicates the number of samples for which the model detects the normal stream sample data as attack stream data. False negative (FN) indicates the number of samples for which the model detects the attack stream data as normal stream data /cite2019SNIPER.

**Figure 6:**
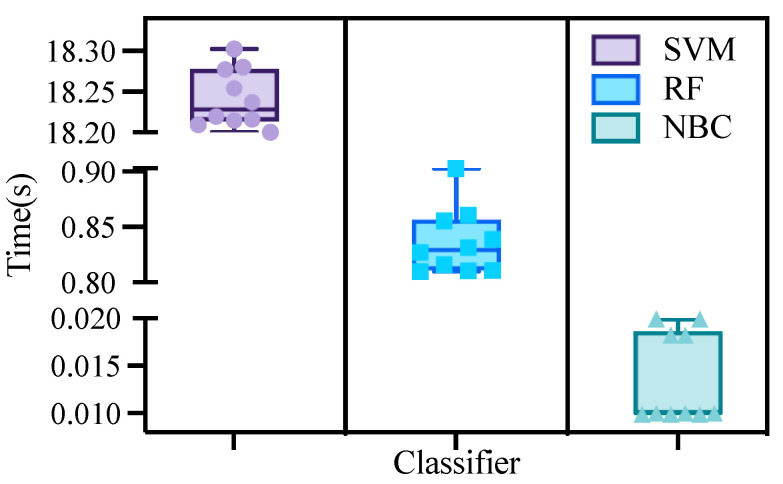
Training time.

The training times of the three algorithms are shown in Figure 6.

Support vector machines required more time to process the data than the Bayesian and random forest methods, with an average time of 18.25 min. The training time for random forest was higher than for Bayes, with an average time of 0.85 min. The Bayesian algorithm took the least time to process the data, with an average time of 0.015 min.

**Figure 7:**
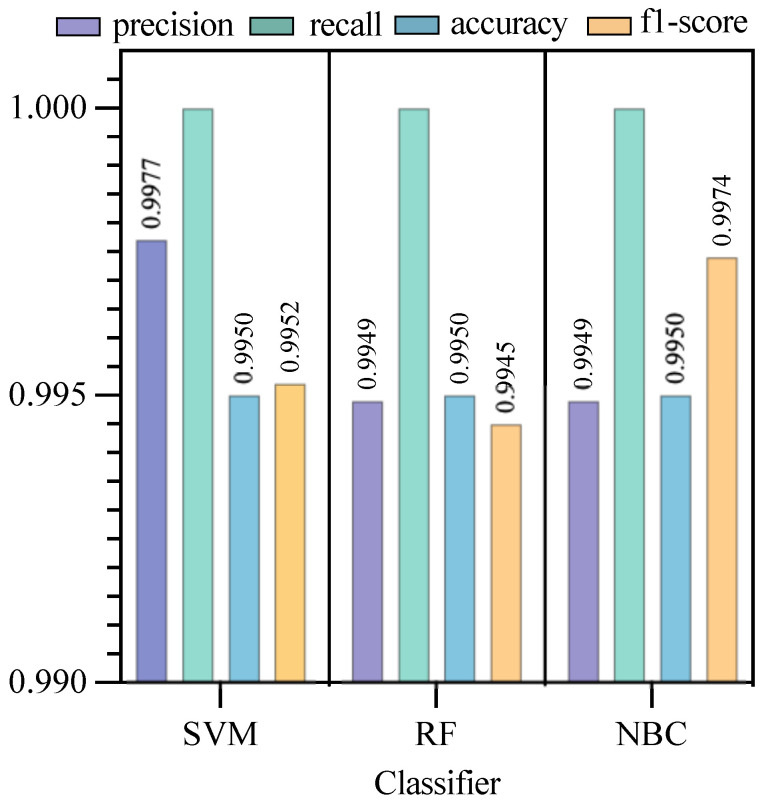
Performance analysis of each type of algorithm.

Based on the above metrics, we experimented with each of the three algorithms and plotted statistics based on their respective metrics, as shown in Figure 7.

The accuracy of support vector machine was 0.9977 and that of random forest was 0.9949. The accuracy of support vector machine was 0.9950 and that of random forest was 0.9950. The F1 score for support vector machine was 0.9952 and that for random forest was 0.9945. Given that the accuracy and precision were similar, the Bayesian algorithm with the higher F1 score was chosen.

By analyzing the data on the training time and various metrics, we find that SVM consumes more time in data processing, while the comparison between RF and NBC is not significant. The difference between RF and NBC in the data on the precision and accuracy is not significant. For this reason, further experiments are needed.

**Figure 8:**
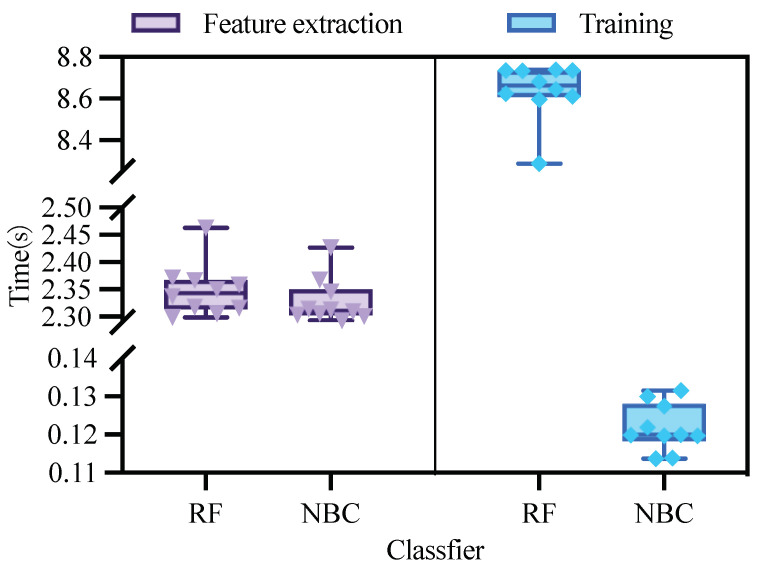
Feature extraction and processing time for RF and NBC.

In the next experiment, the original dataset was compared in terms of both feature extraction time and data processing time by the RF algorithm and NBC algorithm. The feature extraction time and data processing time of the two algorithms are shown in Figure 8. From the figure, we can see that the feature extraction efficiency of NBC is slightly higher than that of RF, but the average time is shorter than that of RF, and both achieve high stability. For the training times of RF and NBC, NBC leads RF, with a faster rate. Therefore, from the above results, the NBC algorithm, with high accuracy, a short training time and a stable training model, is selected in this paper.

Table 5 shows the qualitative analysis of the research on DDoS attacks and defense. The anomaly attack is detected by including the random forest algorithm on SDN [35], but it does not describe the details of the attack. Al-Ani, Ahmed K. [20] studied the protection against DDoS flooding attacks under the address resolution protocol and used the method of address matching to prevent flooding attacks. The Q-learning-based DDoS defense system was introduced in [36] and it uses the colored Petri Net for modeling and simulation. However, it only targets DDoS attacks under IPv4.

## 7. Conclusions

In the future development of IoT, IPv6 will undoubtedly become the specific basis of the IoT communication infrastructure. Therefore, security issues concerning IPv6 must be addressed for IoT network systems. In this paper, we provide an effective protection method to deal with DDoS attacks under the IPv6 communication protocol. In this paper, we propose an NS flooding attack model based on Petri Net and propose a defense detection model based on the NBC algorithm in the SDN framework, supported by a fine-grained analysis of this model. During the experiments, the metrics of the NBC algorithm were analyzed by comparison with other algorithms. The experimental results verify the superiority of NBC against DDoS attacks under IPv6 communication. By analyzing the traffic packets of the detection model in SDN, we found that the controller issues packet drop rules to discard abnormal traffic packets, thus ensuring the normal communication of nodes. In future research, we will model and analyze other attacks of the IPv6 protocol under IoT and analyze the model in real time to study the corresponding detection and defense mechanisms, so as to effectively secure the communication under IoT systems.

## Figures and Tables

**Table 1:** Element mapping relationship table.

Petri Net	NS Flooding
Place	State
Transition	Action
Direct arc	State transform

**Table 2:** Attacking the Place and Transition tables of Petri Net.

Places	Description	Transitions	Description
$P_{0}$	Source node	$T_{0}$	Check the neighbor cache
$P_{1}$	Delay	$T_{1}$	Send NS message
$P_{2}$	Other nodes in the multicast	$T_{2}$	Update the cache table of multicast nodes
$P_{3}$	Obtain the source node IP of the attacker	$T_{3}$	Sniff NS messages
$P_{4}$	Target node	$T_{4}$	Update cache table and send NA messages
$P_{5}$	The source node after receiving the message	$T_{5}$	Send messages
$P_{6}$	Normal state	$T_{6}$	Re-communicate
$P_{7}$	Damaged state	$T_{7}$	Update the neighbor cache table
$P_{8}$	Reachable	$T_{8}$	NS flooding messages
$P_{9}$	Cache table overflow status	$T_{9}$	Receive message

**Table 3:** Table of Place and Transition variations of the defense model.

Places	Description	Transitions	Description
$P_{0}$	Source node	$T_{0}$	Check the neighbor cache
$P_{1}$	Delay	$T_{1}$	Update the cache table of multicast nodes
$P_{2}$	Other nodes in the multicast	$T_{2}$	Send NS messages
$P_{3}$	Forwarding table	$T_{3}$	Send message
$P_{4}$	Controller	$T_{4}$	Send packet-in
$P_{5}$	Flow table with rules	$T_{5}$	Send packet-out
$P_{6}$	Attack machine to obtain source node IP	$T_{6}$	Define rules
$P_{7}$	Hijacking state	$T_{7}$	Sniffing NS messages
$P_{8}$	Normal state	$T_{8}$	Send NS flood messages
$P_{9}$	Hijacked state	$T_{9}$	Less than threshold
$P_{10}$	Reachable	$T_{10}$	Naïve Bayes classification model
$P_{11}$	Detection status	$T_{11}$	Larger than threshold
$P_{12}$	Target node	$T_{12}$	Re-communicate
		$T_{13}$	Update cache table

**Table 4:** All features.

No.	Feature	Description
1	No.	Number
2	Time	Arrive time
3	Source	Source IP
4	Destination	Destination IP
5	Protocol type	Network layer protocol type
6	Checksum	checksum
7	ICMPv6 Type	ICMPV6 Type
8	ICMPv6 Code	ICMPV6 Code
9	Traffic class	Traffic mark field of ICMPV6
10	Next header	Next header field of ICMPV6
11	Hop limit	ICMPV6 hop limit field
12	Ra CHL	RA’s current hop limit field
13	Payload length	Packet payload
14	Flow label	IPv6 traffic label
15	ICMPV6 OPTION	ICMPV6 option type field
16	Ra flag	RA’s flag field
17	RL Ra	RA’s router lifetime field
18	RH Ra	RA’s reachable time field
19	RT Ra	Returns timer field
20	Tadd NS	Target address field of NS
21	Packet size	Icmpv6 data size
22	Sequence	Sequence field of the echo of icmpv6
23	Tadd Na	NA’s target address field
24	Na flag	The flag field of NA

**Table 5:** Model comparison analysis.

Article	State Classification	Formal Analysis	Classification Algorithm	Patched Model	Automatic Operation	IPv6
Tian et al. [35]	×	×	*√*	×	×	×
Al-Ani et al. [20]	×	×	×	×	×	*√*
Feng et al. [36]	*√*	*√*	*√*	*√*	*√*	×
This work	*√*	*√*	*√*	*√*	*√*	*√*

## Data Availability

The data in this article were collected by ourselves. H3 performed an NS flooding attack on H1 using the THC-IPv6 tool, and later captured the information using the Wireshark tool. If the reader would like to access the data in this paper, they may visit www.kaggle.com/hanyu9337/ns-flooding-dataset, accessed on 11 October 2022.
